# Unpaid work and access to science professions

**DOI:** 10.1371/journal.pone.0217032

**Published:** 2019-06-19

**Authors:** Auriel M. V. Fournier, Angus J. Holford, Alexander L. Bond, Margaret A. Leighton

**Affiliations:** 1 Coastal Research and Extension Center, Mississippi State University, Biloxi, Mississippi, United States America; 2 Institute for Social and Economic Research, University of Essex, Colchester, United Kingdom; 3 Ardenna Research, Milton Keynes, United Kingdom; 4 School of Economics & Finance, University of St. Andrews, St. Andrews, United Kingdom; Iowa State University, UNITED STATES

## Abstract

Unpaid work in the sciences is advocated as an entry route into scientific careers. We compared the success of UK science graduates who took paid or unpaid work six-months after graduation in obtaining a high salary or working in a STEM (Science, Technology Engineering and Mathematics) field 3.5 years later. Initially taking unpaid work was associated with lower earnings and lower persistence in STEM compared with paid work, but those using personal connections to obtain unpaid positions were as likely to persist in STEM as paid workers. Obtaining a position in STEM six months after graduation was associated with higher rates of persistence in STEM compared with a position outside STEM for both paid and unpaid workers, but the difference is considerably smaller for unpaid workers. Socio-economic inequality in the likelihood of obtaining entry in STEM by taking an unpaid position is a well-founded concern for scientific workforce diversity.

## Introduction

On 26 April, 2018, the UK House of Lords passed the Unpaid Work Experience (Prohibition) Bill, paving the way for new legislation against unpaid work [1,2]. The new proposed law would prohibit unpaid work exceeding four weeks in duration. The motivation behind this legislation is one of levelling the playing field: in the words of the proposer Lord Holmes, the bill is about “empowerment, enablement, fairness, equality, dignity, respect and talent” [3]. This motivation is based–implicitly–on a three-part reasoning: first, that unpaid work may be exploitative; second, that economic background is a key determinant of access to unpaid work; and third, that unpaid work offers a special leverage into well-paid or desirable careers.

With growing policy concern over the lack of diversity in science [4,5], career steps which present differentially high barriers to young people according to their socioeconomic background deserve particular scrutiny. The Royal Society has established that, in the UK, socio-economic background is a strong predictor of entering the scientific workforce; gender and ethnicity, while also relevant, present a more complex relationship with science careers [6]. While to our knowledge there has been no formal investigation into the backgrounds of unpaid interns in STEM (Science, Technology, Engineering and Mathematics), the case that financial barriers into such positions exist is straightforward. In an influential policy note, the Sutton Trust (a UK foundation for social mobility research and advocacy) estimated that an unpaid intern working in London would incur out-of-pocket expenses of £1,019/month for travel and/or accommodation [7]. Evidence that financial barriers can also affect individuals differently based on gender and ethnicity [8–15] further highlights the potential distortions that unpaid work experience can introduce into the scientific labour market.

The third assumption behind the Unpaid Work Experience (Prohibition) Bill, that unpaid work confers a special advantage in future employment, is more difficult to establish. While anecdotal evidence suggests that young people choosing unpaid work feel that this is a critical step in their career [16–21], and advice and resources targeted at students in science supports this message [21,22] it is unclear to what extent this perception reflects the current labour market in the UK.

Our objective was to bring new evidence to the debate on unpaid work: first, by profiling university science graduates in the UK who were working in unpaid positions six months after obtaining their degrees, and second, by comparing the early career outcomes of those taking unpaid positions after graduation, with those who were in paid work. With the number of graduates doing unpaid work following their degree rising in the UK [22], recently graduated unpaid workers in science jobs are of particular policy relevance.

## Methods and materials

### Data

Our data are from the Destination of Leavers from Higher Education (DLHE) survey administered by the UK Higher Education Statistics Agency (HESA). The population of graduates (from all levels of study) from UK Higher Education Institutions were sent a survey each year to be completed on a specified ‘snapshot day’ in January, approximately six months after leaving university. This obtained an annual cohort of between 214,000–271,000 respondents for graduates between 2003 and 2012, or approximately 80% of the population. A subset of those who responded to the six-month survey and who graduated in 2003, 2005, 2007 or 2009, were contacted for a follow-up survey to be completed on a snapshot day in January approximately three and a half years after graduation, with non-white graduates oversampled at this stage. This follow-up survey obtained an annual cohort of between 17,000–44,000 respondents for the 3.5-year survey, or between 8% (2003 cohort) and 18% (2009) of the population of the relevant cohorts. We restrict our analysis to science graduates (see “Sample Selection” below). These comprised between 20–22% of responses. To account for differential sampling and response probabilities, in our analysis of longer-term (3.5 year) outcomes, we weight the respondents to the 3.5-year survey to the profile of respondents to the 6-month survey by demographic characteristics, degree performance, and characteristics of their initial job (see “Statistical Models” section below).

The surveys collected information about the graduate’s labour market activity or further study being undertaken on the snapshot day, including employment status, the job title, description and industry (from which HESA derives an occupation and industry code according to the Office for National Statistics’ classifications), annual before-tax salary, and contract type. The six-month survey collected information in greater detail on how the current position was obtained and the graduates’ motivation for taking it, while the 3.5-year survey requested retrospective information on previous unemployment and job spells. These were linked to information on the student’s university records, including the institution attended and final degree subject area and classification, and from their application to university (which for domestic students in the UK was managed centrally by the Universities and Colleges Admissions Service, UCAS), which captures information on their parents’ (for ‘young’ students entering university aged 21 or less) or own (for ‘mature’ students) socioeconomic status (likewise based on reported occupation) at time of application to university.

### Ethics

The data were analysed in compliance with the terms of the license between the Higher Education Statistics Agency and the University of Essex (S1 and S2 Appendices), which has been maintained and renewed for the lifetime of the project. Access to the raw data was restricted to the named researcher (Holford), and only in anonymised form.

Consent was obtained at the data collection stage: graduates were informed that data in submitted responses would be linked to existing information held about their degree performance, that this linked survey-administrative data may be used (among other purposes) for “academic research… into higher education where this is of benefit to public interest”, and that anonymised data for these purposes is supplied by HESA to higher education institutions and academic researchers.

Ethical approval for the project ‘Inequality in Higher Education Outcomes in the UK: subjective expectations, preferences and access to information’ was obtained from the University of Essex’s Social Science Faculty Ethics Sub-Committee (S1 Appendix).

### Sample selection

We used data from the six-month surveys for the cohorts leaving university between 2003 to 2012, and from the 3.5-year surveys, linked to the graduate’s earlier response, for the cohorts graduating in 2003, 2005, 2007 and 2009. We restricted our sample to British domiciled British citizens, because information for others was incomplete. We retained graduates of all ages, and all those completing their first Bachelor’s degree. We restricted our sample to those graduating in a science subject, which we defined as one of the following five out of the 19 subject areas: Biological Science (10.4% of all graduates); Agriculture & related subjects (0.8%), Physical sciences (5.0%); Mathematical sciences (2.0%); and Computer sciences (4.7%). Finally, in our main regression analyses, we restrict the sample to those in paid or unpaid work 6 months after graduation, in paid work 3.5 years after graduation (for the long-term outcome regressions), whose household SES was collected (even if it could not be classified), and for whom information on the relevant job characteristics was available.

### Derived variables

#### Explanatory

In our analysis we defined ‘unpaid work’ as reporting employment circumstance six months after graduation as being in ‘voluntary work or other unpaid work’ (9,180 science graduates, 1.8% of the total). This included 1,010 graduates whose primary activity was undertaking “Work and further study” but for whom the work component was unpaid. (Sample sizes and degrees of freedom are rounded to the nearest 5, to meet the disclosure requirements of the data providers). The comparator group was ‘In Work’, i.e. in full or part-time paid work, or self-employment. Note that we do not assess the legality of individuals’ positions under the current legislation. (The key principle is that the unpaid worker should not be substituting for a paid worker, though extensive guidance and examples on exemptions for voluntary work and expenses-only arrangements are provided for employers and workers on the UK government website [23]).

Additional explanatory variables of interest were:

Socioeconomic status (SES): ‘High SES’ individuals were those whose parents’ (or if aged over 21, own) occupation *at time of applying to university* was classified as “Higher Managerial or Professional” or “Lower Managerial and Professional”, categories 1 and 2 in the National Statistics Socio-economic Classification (NS-SEC). ‘Low SES’ included categories 3 (‘Intermediate occupations’) to 7 (‘Routine occupations’) and 8 (‘Never worked and long-term unemployed). SES was not classifiable from the job title submitted to UCAS for 21.5% of science graduates. In our tables comparing High and Low SES individuals we omitted those whose SES was not classifiable. In our regression models we retained these individuals and include a dummy variable for ‘SES not classified’.

Legal sex at birth: There were slightly more male (51.6%) students than female (48.4%), using legal sex assigned at birth.

‘Good Degree’: For those obtaining a first undergraduate degree, whether they received a ‘Good’, First or Upper second-class degree or a ‘Lower’, lower second or third-class degree. A First or Upper Second is usually the minimum necessary for most graduate jobs in the private sector, civil service, or progression into a Master’s degree. Typically, the degree class is what will be reported on a graduate’s CV/resume, though employers could ask for a detailed transcript. In our tables comparing Good and Lower degrees we omitted those whose degree class was not classified (2.5%). In our regression models we retained these individuals and included a dummy variable for ‘degree class not classified’.

Personal connections: Those in employment were asked ‘How did you find out about this job?’, and were allowed to select one option. We deemed those choosing “Personal contacts, including family and friends, networking” to have found their position through personal connections.

The means of all the covariates, having applied these sample restrictions, are shown in Table 1.

**Table 1:** Covariate means for main regression specifications.

Sample proportions, %	6 months (unweighted)	3.5 years (weighted)
*Demographics*:		
High SES	42.46	42.03
SES Not classified	21.55	21.72
Female	49.83	47.96
Good Degree	61.86	60.59
Degree class unclass’	2.46	2.55
White British	85.15	86.79
Black Brit’ inc.mixed	2.46	1.91
Asian Brit’ inc.mixed	8.23	7.28
Other ethnicity	4.15	4.02
*Characteristics of job 6 months after graduation*:		
Unpaid work	2.97	2.57
Found through personal connections	19.06	18.87
In STEM	24.07	25.31
Unpaid *and* Personal connections	0.83	0.77
Unpaid *and* in STEM	0.62	0.51
N	207,220	10,545

#### Outcomes

All of the outcome variables were taken from the 3.5-year follow-up survey.

Ln salary: Conditional on being in work, graduates reported their annual gross pay, before tax, from which HESA derived an annual measure. This was missing for approximately 11% of science graduates in work. For these item-non-respondents we imputed the salary as the estimated mean before-tax annual earnings within a cell in the UK Annual Population Survey for the financial year of the relevant DLHE survey, defined by: 3-digit occupation code; industry section; full-time/part-time; government office region; 3 categories for number of employees in the organization; permanent contract. We used a Tobit model, accounting for salaries not falling below zero, and those above £40,000 being recorded simply as “above £40,000”. This figure was then converted to January 2013 levels for all years using the Retail Price Index from the Office for National Statistics, before taking the natural logarithm.

Science jobs: We defined science, technology, engineering, mathematics (STEM) occupations using a derivation of the classification compiled by the Royal Society [6]. In the UK, jobs were classified by the Office for National Statistics (ONS) into the Standard Occupational Classification (SOC). The Royal Society used the most detailed codes available to map occupations as STEM, Possibly STEM, and non-STEM. The DLHE classifies occupations at a coarser level. We defined a respondent’s job as ‘definitely STEM’ if all the possible jobs that the occupation listed in the DLHE could represent were classified as STEM.

### Statistical models

We present estimates obtained using the following statistical methods.

**Fig 1:**
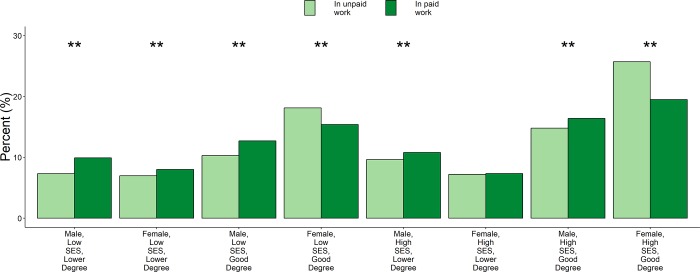
Composition of Science Graduates in paid and unpaid work 6 months after graduation. Unpaid workers are disproportionately women with good degrees, regardless of SES class. Note: Sample includes those who completed the survey 6 months after graduation, had complete records of SES and degree class, and were in paid or unpaid work. Stars indicate: * = <0.05; ** = <0.01 for two-sample test of differences in proportions.

For univariate comparisons of the characteristics of science graduates selecting into unpaid versus paid work after 6 months (see Table 2, and indicators of statistical significance in Fig 1) we employ a two-sample test of differences in proportions. The formal specification of this test is shown in S1 File.

**Table 2:** Characteristics of science graduates in paid and unpaid work 6 months after graduation.

All per cent	All		Complete SES and Degree class information	
	Unpaid work	Paid work	Unpaid work	Paid work
Female	58.0	48.8	58.3	50.3
*Diff*	9.2**		8.0**	
*Std error*	(0.58)		(0.72)	
High SES	37.8	31.8	57.7	54.0
*Diff*	5.9**		3.7**	
*Std*. *error*	(0.57)		(0.72)	
Low SES	27.7	27.1	42.3	46.0
*Diff*	0.6		-3.7**	
*Std*. *error*	(0.53)		(0.72)	
SES not classified	18.3	16.2		
*Diff*	2.1**			
*Std*. *error*	(0.46)			
Good Degree	66.1	60.6	68.7	63.5
*Diff*	5.5**		5.2**	
*Std*. *error*	(0.56)		(0.68)	
Degree Class not classified	1.3	2.6		
*Diff*	-1.4**			
*Std*. *error*	(0.13)			
White British	77.9	84.7	80.6	87.6
*Diff*	-6.8**		-6.9**	
*Std*. *error*	(0.48)		(0.006)	
Black British	4.6	2.4	3.7	2.0
*Diff*	2.2**		1.8**	
*Std*. *error*	(0.24)		(0.27)	
Asian British	11.8	8.7	10.8	7.0
*Diff*	3.1**		3.8**	
*Std*. *error*	(0.38)		(0.45)	
Other Ethnicity	5.6	4.2	4.8	3.4
*Diff*	1.4**		1.4**	
*Std*. *error*	(0.27)		(0.31)	
N	7,340	267,680	4,805	157,760

Stars indicate

* p<0.05

** p<0.01

from two-sample test of differences in proportions

**Fig 2:**
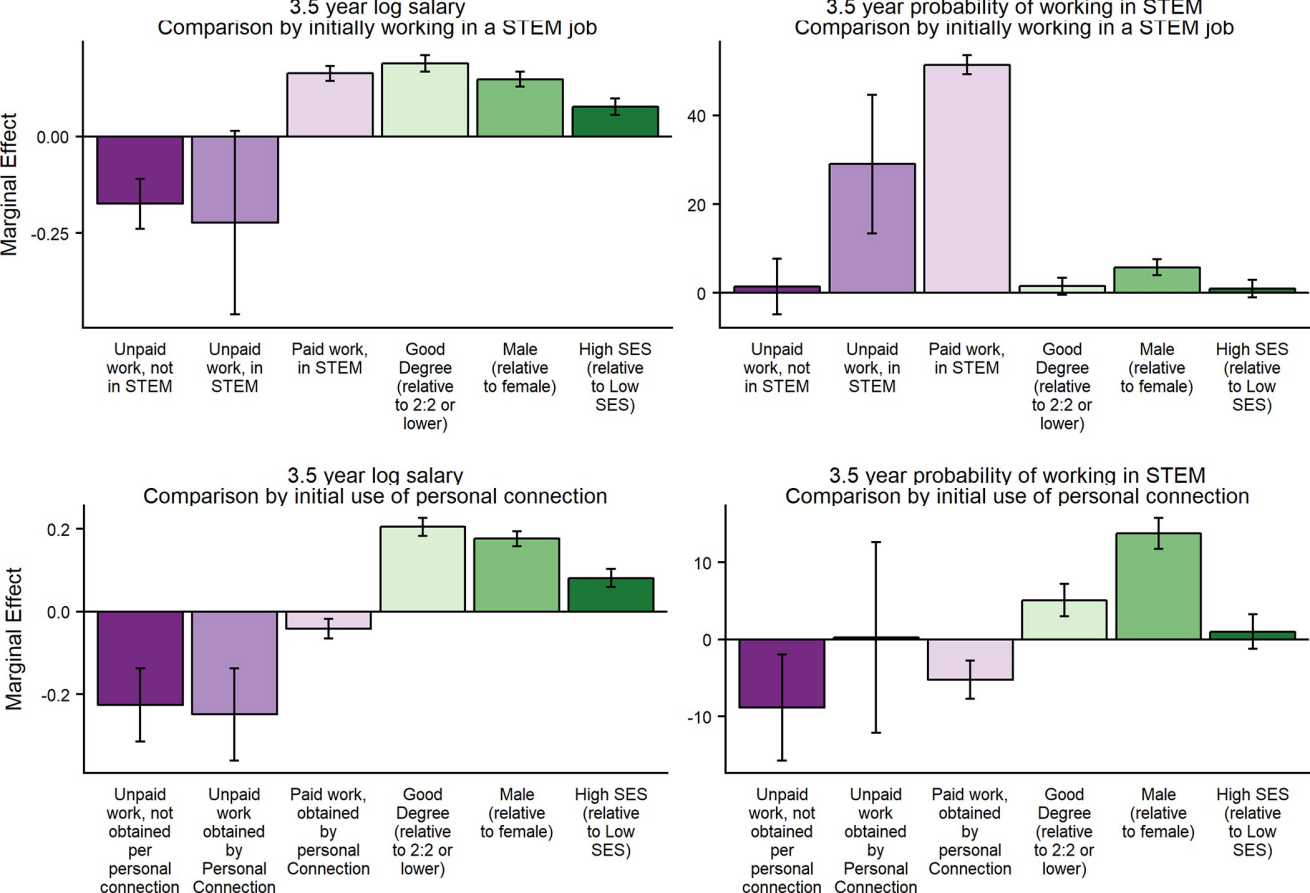
Multivariate regression analysis of earnings and persistence in STEM. Top panels: Conditional marginal differences are compared to ‘Paid Work at 6 months, in STEM’. Relative to paid work not in STEM, whether an unpaid position is in STEM or not is conditionally uncorrelated with salaries, but for paid workers initially taking a STEM role has a persistent association with salaries. Bottom panels: Conditional marginal differences are compared to ‘Paid work at 6 months, not obtained by personal connection’. Taking unpaid work, regardless of how it was obtained, had a negative association with salary. Taking unpaid work only has a negative association with persistence in STEM when not found through personal connections. Note: Error bars represent 95% confidence intervals for the conditional marginal difference.

For multivariate regression estimates we employ different models, as appropriate to the form of the dependent variable. Where the dependent variable was binary (participation in follow-up survey, Table A in S1 File; 6-month position found by personal connections or is in STEM, Table 3; 3.5-year position is in STEM, Fig 2 and Table B in S1 File) estimates were obtained using ordinary least squares regression. For our main outcome regression of interest; whether the individual was working in a science job after 3.5 years the estimated equations took the form:

$${\mathrm{SJ}}_{i}=\alpha_{0}+\alpha_{1}int_{i}+\alpha_{2}{jobchar}_{i}+\alpha_{3}({unpaid}_{i}*{jobchar}_{i})+\alpha_{4}male_{i}+\alpha_{5}SES_{i}+\alpha_{6}{deg}_{i}+\epsilon_{i}$$ (1)

where *SJ*_*i*_ was a dummy variable equal to one if the individual was working in a science job 3.5 years after graduation. *unpaid*_*i*_ was a dummy variable equal to one if the individual was doing unpaid work six months after graduation; *jobchar*_*i*_ is a dummy variable equal to 1 if the six month position met a particular characteristic (either being found by personal connections, or initially being in a STEM field); *male*_*i*_ was a dummy variable equal to 1 for individuals who report their sex as male; ***deg***_***i***_ was a vector of dummy variables equal to 1 if the individuals’ undergraduate degree was either a ‘good’ class (First or Upper Second) or ‘not classified’ (the omitted category was a lower class degree); ***SES***_***i***_ was a vector of dummy variables equal to one if the individual was from a high SES household, or where this was not classified (the omitted category was a low SES household). The coefficients to be estimated were *α*_1_−*α*_6_, (reported in Table B in S1 File) with linear combinations of *α*_1_−*α*_3_ forming the basis of the conditional marginal differences reported in Fig 2. (For example, the conditional predicted difference in the outcome for an unpaid worker finding their position through personal connections compared with a paid worker finding their position through other means will be (*α*_1_+*α*_2_+*α*_3_)).

**Table 3:** Multivariate regression of whether six-month position was found through personal connections or was in STEM, on demographic and job characteristics.

Dependent variable: →	Six-month position found through personal connections			Six-month position was in a STEM field		
	(1)	(2)	(3)	(4)	(5)	(6)
Sample →	All workers	Unpaid workers only	Paid workers only	All workers	Unpaid workers only	Paid workers only
Explanatory variables ↓						
Unpaid Work	0.093**			-0.015**		
	(0.005)			(0.005)		
Female	-0.019**	-0.035**	-0.019**	-0.153**	-0.051**	-0.156**
	(0.002)	(0.011)	(0.002)	(0.002)	(0.011)	(0.002)
High SES	0.010**	0.016	0.010**	-0.003	0.002	-0.003
	(0.002)	(0.012)	(0.002)	(0.002)	(0.012)	(0.002)
Good Degree	-0.019**	-0.005	-0.019**	0.088**	0.066**	0.088**
	(0.002)	(0.012)	(0.002)	(0.002)	(0.011)	(0.002)
SES not classified	-0.003	-0.027	-0.003	0.016**	0.002	0.016**
	(0.002)	(0.015)	(0.002)	(0.003)	(0.015)	(0.003)
Degree class not classified	-0.014*	-0.040	-0.014*	0.027**	-0.007	0.027**
	(0.006)	(0.053)	(0.006)	(0.006)	(0.049)	(0.006)
Ethnicity dummies	Yes	Yes	Yes	Yes	Yes	Yes
Cohort Dummies	Yes	Yes	Yes	Yes	Yes	Yes
N	221,765	6,905	214,860	207,220	6,150	201,070

Stars indicate

* p<0.05

** p<0.01

Where the dependent variable was *the natural logarithm of earnings* we used a Tobit regression which allowed for truncation of the dependant variable. The model’s lower bound was set at £0, while the upper bound set to £40,000, the range over which we have full information with which to impute missing salaries from the Annual Population Survey. The estimated equations took the form:

$$(\ln\;y_{i}{)}^{*}=\beta_{0}+\beta_{1}int_{i}+\beta_{2}{jobchar}_{i}+\beta_{3}(int_{i}*{jobchar}_{i})+\beta_{4}male_{i}+\beta_{5}SES_{i}+\beta_{6}{deg}_{i}+\epsilon_{i}$$ (2)

$$\epsilon_{i}∼N(0,\sigma^{2})$$

$$\ln\;y_{i}=(\ln\;y_{i}{)}^{*}\;if\;(\ln\;y_{i}{)}^{*}<10.5966$$

$$\ln\;y_{i}=if\;(\ln\;y_{i}{)}^{*}\geq 10.5966$$

Here, (ln *y*_*i*_)* was a latent continuous variable representing the ln of individual earnings measured 3.5 years after graduation; ln *y*_*i*_ was the ln of *observed* individual earnings, which were censored at £40,000 per year. The coefficients to be estimated were *β*_1_−*β*_6_ (reported in Table B in S1 File) with linear combinations of *β*_1_−*β*_3_ forming the basis of the conditional marginal differences reported in Fig 2.

All sample statistics and regression models addressing outcomes measured 3.5 years after graduation are estimated with observations weighted to the profile of the respondents to the 6-month survey. That is, we weight in inverse proportion to their predicted probability of participation in the survey at 3.5 years conditional on having participated at 6 months, from the probit regression model presented in Table A in S1 File. This enables us to use all the available observations for our analysis while accounting for the combination of deliberate over-sampling of some demographic groups and differential response rates to the 3.5-year follow-up survey. The sample proportions of the covariates (all dummy variables) are shown in Table 1. The first column shows those in paid or unpaid work after 6 months. The second column shows the weighted proportions of those observed again 3.5 years after graduation, after excluding those not in paid work at that time. These form the estimation sample for Eqs (1) and (2).

### Data and materials availability

This work uses the following data provided by the Higher Education Statistics Agency (HESA), all copyright Higher Education Statistics Agency Limited 2013:

- HESA Destination of Leavers survey 2002/03-2011/12
- HESA Destination of Leavers Longitudinal Survey 2002/03–2008/09.

HESA cannot accept responsibility for any inferences of conclusions derived from the data by third parties. The data used in this paper were analysed under license from HESA. The terms of this license prevent us from sharing the data directly with members of the public or other researchers, as the large set of variables included in the data and the population sample frame create a high risk of disclosure of confidential information about individual respondents. However, the same extracts can be obtained directly from HESA by applying with the same specification, available in S2 Appendix. Stata code for analyses (S1 Code) and R code for figure creation (S1 Fig, S2 Fig, S3 Fig) are also included in the supporting information. The use of the data in this work does not imply the endorsement of any organization in relation to the interpretation or analysis of these data.

## Results

We used data from surveys of 2003–2012, with the six-month survey comprising responses from 1,932,775 graduates from Bachelors’ degrees (sample sizes and degrees of freedom are rounded to the nearest 5, to meet the disclosure requirements of the data providers). The follow-up survey carried out for the 2003, 2005, 2007 and 2009 cohorts, included 98,250 graduates. 442,335 of the first wave survey respondents, and 25,755 of the follow-up respondents, graduated with a degree in one of the following subject areas as defined by the Higher Education Statistics Agency (HESA): Biological Sciences, Agriculture and Related Studies, Physical Sciences (which includes Chemistry), Mathematical Sciences, or Computer Sciences. We retained only these science graduates in our analysis. For our regression analyses of the associations between participation in unpaid work and subsequent labour market outcomes, we further restrict to those either in paid or unpaid work 6 months after graduation (275,010 observed at 6 months, 16,110 observed at 3.5 years), and for pairwise comparisons by SES or degree class, to those with complete information on these variables (i.e. no “unclassified”; 162,560 and 10,150 respectively). As robustness checks, we re-estimated our main specifications excluding first mature students (whose SES is based on their own pre-university occupation rather than their parents’ occupation) and then those whose 3.5 year salary required imputation. These are shown in Tables C and D in S1 File. These introduce no significant or quantitatively important changes in personal characteristics associated with job characteristics 6-months after graduation, or between job characteristics 6 months and 3.5 years after graduation.

Table E in S1 File shows the characteristics of this population of science graduates; those responding to the 6-month survey (for which the entire population was targeted); and those responding to the 3.5-year survey (for which only a subset of the 6-month respondents were targeted for participation). It is not possible in our data extract to differentiate between those not selected for participation at 3.5 years and those not responding, but the net result is to produce a sample containing a higher proportion of high SES graduates, high degree performers, women, ethnic minorities, and (conditional on being in paid or unpaid work after 6 months) those initially in a STEM field or a position found through personal connections. There was no difference in follow-up sample response by initial participation in unpaid work. In Table A in S1 File we present OLS and probit regression coefficients for predictors of responding at 3.5 years, conditional on responding and being in paid or unpaid at 6 months. The predicted probabilities from the probit regression are used to derive inverse probability weights for sample statistics and regressions on outcomes measured after 3.5 years.

### Who takes unpaid work?

Six months after graduation, most science graduates were in paid work (64%) while 2% were in unpaid work, 20% were taking further study, and 15% doing something else (Table F in S1 File). Women and those known to be high SES or have a good degree, were significantly more likely to be taking unpaid work than men, or those known to be low SES or to have a lower degree respectively (two-sample tests of differences in proportions shown in Table F in S1 File). Table 2 shows the composition of those in unpaid and paid work by demographic characteristics and degree performance. A greater proportion of unpaid than paid workers were female (58.0% vs. 48.8%), known to be high SES (37.8% vs. 31.8%), and known to have secured a good degree (66.1% versus 60.6%). The magnitude and significance of these differences remains unchanged when excluding those with unclassified SES or degree class.

Fig 1 shows the breakdown of those in paid and unpaid work by SES, sex and degree class. High SES women with Good Degrees were especially over-represented in unpaid work (25.7% in unpaid vs 19.5% in paid work, *diff* = 6.22%, SE = 0.60%, *p* < 0.0001).

### How do former unpaid workers compare paid workers, three years later?

We now restrict our sample to those in unpaid work or paid work 6 months after graduation and who responded to the follow-up survey, and weight these to the profile of all science graduates observed 6 months after graduation. 84.5% were in paid work, 8.8% in further study, 6.2% doing something else, and 0.4% in unpaid work.

Those working earned a mean annual salary of £26,130 (all salaries are expressed before tax, and in January 2013 British pounds), and 32.8% were working in STEM, according to the list of occupations defined by the Royal Society as “definitely” part of the “scientific workforce” [3].

Science graduates who were in unpaid work six months after graduation and paid work after 3.5 years had a significantly lower annual income at the later point, compared to those who initially took paid work, but they were not significantly less likely to be working in STEM. After controlling for degree class, sex, SES, ethnicity, and cohort of graduation in a Tobit regression, graduates who had initially taken unpaid work, but three years later were in paid employment, had 22.5% lower gross salary compared with graduates who had initially taken a paid position (*p < 0*.*001*, Table B in S1 File). After controlling for degree class, sex, SES, and cohort of graduation in a linear regression, science degree holders who were doing unpaid work six months after graduation and were in paid work 3.5 years after gradation were 5.1 percentage points less likely to be employed in a STEM occupation than graduates who were initially in paid work, but this difference was not statistically significant (*p =* 0.106, Table B in S1 File).

All else equal, there were large differences in outcomes between sexes, with men earning 17.5% higher salaries (*p* < 0.001) and being 13.6 percentage points more likely to be in a STEM job 3.5 years after graduation than women (*p* < 0.001); and by degree class, with good degree holders earning 20% higher salaries (*p* < 0.001) and 5.2 percentage more likely to be in a STEM job (*p* < 0.001) than lower degree class holders. High SES graduates earned 8.8% higher (*p* < 0.001) but were no more likely to be in the scientific workforce after 3.5 (*p* = 0.361).

### Are some unpaid positions better than others?

**Fig 3:**
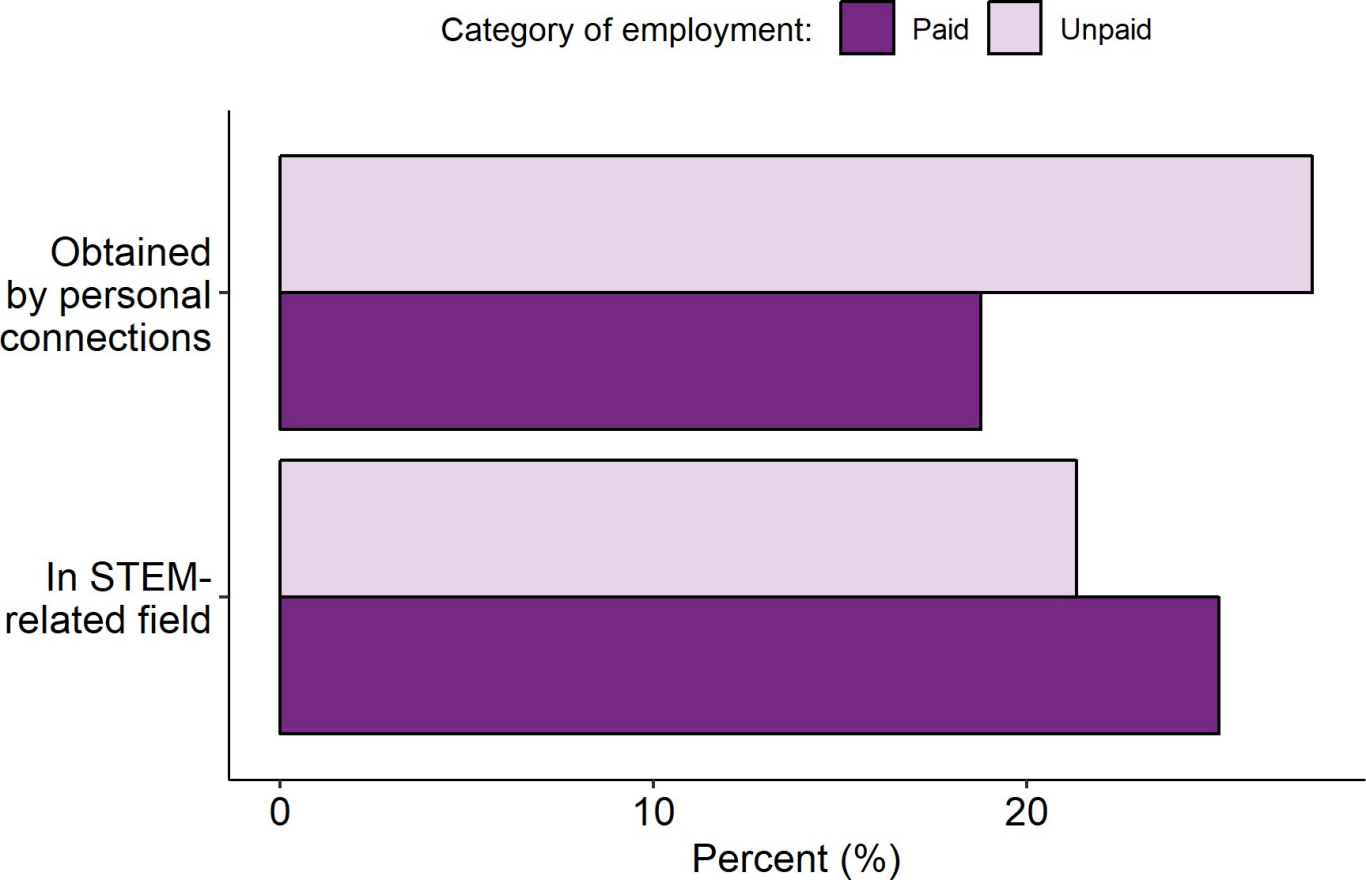
Characteristics of paid and unpaid work among science graduates six months after graduation. Unpaid work was found more frequently through personal connections than paid work, while a greater proportion of paid workers were in a STEM field.

The DHLE has limited information with which to compare the occupations of different respondents. We considered two characteristics which might reflect the quality of a position: first, how the respondent found their six-month position; second, whether or not the position was in STEM. We next describe the frequency of these characteristics across paid and unpaid work (Fig 3); the individual characteristics which predict these job characteristics 6 months after graduation and whether this differs for paid and unpaid work (Table 3); and whether outcomes three years later differed according to these job characteristics in the 6-month position (Fig 2; Table B in S1 File).

### Positions found using personal connections versus other means

As a part of the 6-month survey graduates in paid or unpaid work were asked how they found their position. We grouped these responses into two categories: personal connections (“personal contacts, including family and friends”, 19.0%) and other (which included university careers services, recruitment agencies, employer websites, media advertisements, professional networking, and speculative applications, as well as ‘other’ or no method given, 81.0%). Six months after graduation, a greater share of unpaid workers reported finding their position through personal connections than did paid workers. Controlling for degree class, sex, SES, ethnicity, and the cohort of graduation, unpaid workers were 9.3 percentage points more likely to have found their position at six months through personal connections than paid workers (*p* < 0.001; Table 3).

The reliance on personal connections varied according to personal characteristics. Pooling those in paid and unpaid work, women were 1.9 percentage points less likely to have found their position using personal connections (*p* < 0.001; Table 3), while graduates from lower SES families were 1 percentage point less likely to have done so, compared with graduates from High SES families *p* < 0.001; Table 3), and graduates with lower class degrees were 1.9 percentage points more likely to have done so than graduates with Good Degrees (*p* < 0.001; Table 3). The sex difference is especially large for those in unpaid work: women were 3.5 percentage points less likely to have found their unpaid position through personal connections (*p* < 0.001; Table 3) than men.

Unpaid workers who found their 6-month position through personal connections have very similar earnings three years later to unpaid workers who found their 6-month position by other means. These earn 24.9% and 22.6% less than the base category of former paid workers who did not find their position through personal connections (Fig 2, p-value of both conditional marginal differences <0.001, p-value of difference = 0.740). In contrast, paid workers who found their 6-month position through personal connections earned 4.2% less, three years later, than those who were in paid work found through other means (*p* = 0.001; Fig 2; Table B in S1 File).

Those former unpaid workers who found their position through personal connections were 9.1 percentage points more likely to be working in STEM occupations three years later, compared with their unpaid peers who found their position in other ways. This difference is not statistically significant (p = 0.205), but the sign and magnitude of the point estimate contrasts with paid workers who found their 6-month position through personal connections, who are 5.3 percentage points *less* likely to be working in STEM three years later than paid workers who found their 6-month position by other means (p<0.001).

This means that despite the significant negative association overall between unpaid work and persistence in STEM, unpaid workers who found their position through personal connections are in fact almost equally likely to be working in STEM three years later as paid workers who found their position through other means (difference is 0.2 percentage points, *p* = 0.970).

### Is unpaid work in science better than paid work in another field?

Six months after graduation, 25.1% of those in paid or unpaid work were in a STEM occupation. Controlling for degree class, sex, SES, and cohort of graduation, unpaid workers were 1.5 percentage points less likely to be working in STEM than paid workers (p<0.001; Table 3). Pooling those in paid and unpaid work six months after graduation, women were 15.3 percentage points less likely to be working in STEM (*p* < 0.001). Graduates from Low SES families were not significantly less likely to be working in STEM than graduates from High SES families (*p* = 0.172; Table 3). Graduates with lower class degrees were 8.8 percentage points less likely to be working in STEM than graduates with Good Degrees (*p* < 0.001; Table 3).

Compared with those graduates whose six-month unpaid position was not in STEM, former unpaid STEM workers did not have significantly different earnings three years later (4.86% less, *p* = 0.699; Fig 2; Table B in S1 File). A contrasting pattern holds between paid workers whose six-month position was in STEM and non-STEM: The former had 16.3% higher earnings three years later (*p* < 0.001; Fig 2; Table B in S1 File).

Pooling those in paid and unpaid work six months after graduation, those whose six-month position was STEM-related were 51 percentage points more likely to be working in a science job three years later, compared with those whose initial position was outside STEM (*p* < 0.001; Table B in S1 File). This positive association between being initially and subsequently in a STEM position holds separately for both paid and unpaid workers (51.6 percentage points, *p* < 0.001; and 27.6%, *p* = 0.001 respectively; Fig 2; Table B in S1 File). This means that those whose six-month position was unpaid, but STEM related, were still 29.0 percentage points more likely to persist in STEM than those whose six-month position was paid, but in a field unrelated to STEM (*p* < 0.001; Fig 2; Table B in S1 File).

## Discussion

Graduates’ routes into unpaid work, and the field it is in, are strongly associated with subsequent outcomes. Unpaid work found through personal connections was associated with higher rates of persistence in STEM, compared with that found in other ways, offsetting the negative overall relationship between unpaid work and persistence in STEM. Personal connections were more frequently used to find unpaid work by men, high-SES graduates, and lower degree performers. STEM-related unpaid work was also strongly associated with persistence in STEM three years later, compared with work in other occupations. We found a negative association between doing unpaid work six months after graduation and earnings three years later.

While this evidence failed to support the hypothesis that unpaid work plays a prominent role in securing high-paying positions for recent graduates, it suggests that taking unpaid work in a STEM position can act as a stepping stone towards accessing future paid STEM professions. For recent science graduates who do not secure paid work in STEM and face a choice between staying in science and working for free, or leaving science and taking a paid position, doing the former is a rational decision for those who have the resources or (especially) connections to do so, and who wish to remain in STEM.

### Limitations

Our analysis allows us to explore the association between various individual and job characteristics. It is important to note, however, that these associations cannot be given a strong causal interpretation. The relatively small number of young people taking unpaid positions, and the diversity of motivations behind this choice–from a lack of better alternatives, to the passionate pursuit of a particular line of work–makes drawing causal inference on the impact of unpaid work on future career outcomes not possible with these data. Unmeasured or unobservable characteristics of those who took unpaid and paid work are likely to be strong determinants of future career outcomes.

It is also important to note that our analysis is based on science graduates, and we were unable to assess barriers that arose prior to university graduation that take members of underrepresented groups away from scientific careers or prevent them from finishing a degree. Barriers to access in science begin early: by age 13, many students from underrepresented groups have already been discouraged from their interest in science [23,24]. This disparity continues throughout secondary education and has an impact on the composition of the incoming classes of undergraduate science degree programmes [25]. Once students arrive at university, these barriers continue, and new ones are added, from outright discrimination, harassment and assault [26,27], to faculty who more subtly discourage or refuse to support a diverse classroom [28], in addition to unpaid work that can be taken before a student graduates.

It remains possible that certain unpaid jobs are “career-makers”; equally, there may be particular career paths in science which are effectively unattainable without spells of work experience for which paid positions are non-existent or scarce. However, we found no evidence that unpaid work shortly after graduation lead to high-paying jobs several years later: on the contrary, those who were in paid work six months after graduation out-earned those who were in unpaid work at the same time three years later. This raises the concern that those who choose this career path–disproportionately women who have graduated with good grades–are making a career choice that is not only costly in the short term, but which is still associated with lower earnings three years later. Future research should explore in greater depth why high academic-achieving women are overrepresented in such positions, and the contribution this makes towards explaining gender-based wage gaps throughout the career.

Our analysis has shown that work experience in science shortly after graduation is strongly associated with persistence in science occupations three years later. This suggests that, from the perspective of a diverse scientific workforce, the concerns about access to unpaid work based on socioeconomic status are potentially well-founded: this financially-costly career stage could drive young people from disadvantaged backgrounds away from science careers before these careers have properly begun. On the other hand, it also suggests that access to early-career work experience in science is a key determinant of persistence in scientific work. Policies which limit unpaid work, such as the UK’s proposed Unpaid Work Experience (Prohibition) Bill, may have the unintended consequence of reducing opportunities for recent science graduates to remain in science. Alternative policies, which make the acquisition of valuable early-career scientific work experience affordable and accessible to science graduates regardless of their financial ability, could level the playing field while also keeping aspiring scientists in work related to their training.

## Supporting information

S1 Fig R script for recreating Fig 1. (R)

S2 Fig R script for recreating Fig 2. (R)

S3 Fig R script for recreating Fig 3. (R)

S4 Fig Input files for recreating Fig 2. (ZIP)

S1 Code Stata code that produced our results. (ZIP)

S1 File Supplementary Text and Tables. (PDF)

S1 Appendix Ethics approval letter. (PDF)

S2 Appendix Specification to request the same dataset we used. (PDF)
