# Supplementary material for: Unpaid work and access to science professions
Source: PLoS One. 2019 Jun 19;14(6):e0217032. doi: 10.1371/journal.pone.0217032 (PMC6583997; doi:10.1371/journal.pone.0217032)
Supplement: S1 File — (PDF) [file pone.0217032.s006.pdf]

Supplementary Text and Tables

Two-sample test of difference in proportions

Formally, we test the difference between two large samples of size n_1 and n_2 , and with x_1 and x_2 observed successes, in the proportion of observed successes $\hat{p}_1 = \frac{x_1}{n_1}$ and $\hat{p}_2 = \frac{x_2}{n_2}$. Where α is the significance level of interest, and $z_{1-\frac{\alpha}{2}}$ and $-z_{1-\frac{\alpha}{2}}$ are the z-statistics enclosing the proportion α of the inverse cumulative standard normal distribution, the confidence interval for the difference in proportions is given by:

$$CI_\alpha = (\hat{p}_1 - \hat{p}_2) \pm z_{1-\frac{\alpha}{2}} \sqrt{\frac{\hat{p}_1\,(1-\hat{p}_1\,)}{n_1} + \frac{\hat{p}_2\,(1-\hat{p}_2\,)}{n_2}}$$

Alternatively stated, the following test statistic was normally distributed, so where $|z|>\left|z_{1-\frac{\alpha}{2}}\right|$, the null hypothesis of no difference in proportions between the groups can be rejected at the 1-α level:

$$z = \frac{\hat{p}_1 - \hat{p}_2}{\sqrt{\hat{p}_{all}\,(1-\hat{p}_{all})(\frac{1}{n_1} + \frac{1}{n_2})}}$$

Supplementary Tables

The results reported in Figure 3 came from linear combinations (with associated confidence intervals) from four multivariate regressions shown in columns 2, 3, 5 and 6 of Table B, below.

Table A: Modelling participation in the follow-up survey

	All		Complete SES and Degree class information	
	OLS	Probit (Ave. Marginal Effects)	OLS	Probit (Ave. Marginal Effects)
Female	0.008**	0.009**	0.009**	0.009**
	(0.001)	0.001	(0.001)	(0.001)
Low SES	(Base)	(Base)	(Base)	(Base)
High SES	0.005**	0.005**	0.004**	0.004**
	(0.001)	(0.001)	(0.001)	(0.001)
SES not classified	-0.008**	-0.013**	x	x
	(0.001)	(0.001)		
SES not collected	-0.008**	-0.013**	x	x
	(0.001)	(0.001)		
Good Degree	0.011**	0.012**	0.010**	0.011**
	(0.001)	(0.001)	(0.001)	(0.001)
Lower Degree	(Base)	(Base)	(Base)	(Base)
Degree class unclassified	0.009**	0.010**	x	x
	(0.003)	(0.003)		
White	(Base)	(Base)	(Base)	(Base)
Black	0.039**	0.041**	0.013**	0.013**
	(0.003)	(0.004)	(0.004)	(0.005)
Asian	0.012**	0.012**	-0.000	-0.000
	(0.002)	(0.002)	(0.002)	(0.002)
Other	0.039**	0.040**	0.045**	0.045**
	(0.002)	(0.003)	(0.003)	(0.004)
Labour market outcome at 6 months:				
Unpaid work	0.004	0.005	0.004	0.004
	(0.004)	(0.004)	(0.005)	(0.005)
Found through personal connections	0.003**	0.003**	0.003	0.003
	(0.001)	(0.001)	(0.002)	(0.002)
In a STEM field	0.016**	0.015**	0.020**	0.019**
	(0.001)	(0.001)	(0.001)	(0.001)
Unpaid work x Found through personal connections	-0.007	-0.007	-0.004	-0.004
	(0.006)	(0.006)	(0.008)	(0.008)
Unpaid work x In a STEM field	-0.009	-0.009	-0.008	-0.008
	(0.007)	(0.006)	(0.009)	(0.008)
N	275,020	275,020	162,560	162,560
Dependent variable mean	0.059	0.059	0.063	0.063

Stars indicate: * $p<0.05$, ** $p<0.01$

Table B: Multivariate regression of labour market outcomes 3.5 years after graduation on demographic and job characteristics 6 months after graduation.

	Outcome 3.5 years after graduation					
Dependent variables→	Real ln salary[1]			In STEM workforce		
	(1)	(2)	(3)	(4)	(5)	(6)
	Overall	By use of personal connect's	By initial STEM occupation	Overall	By use of personal connect's	By initial STEM occupation
Unpaid work at 6 months	-0.225**	-0.226**	-0.179**	-0.051	-0.089*	0.013
	(0.036)	(0.045)	(0.033)	(0.031)	(0.035)	(0.032)
Personal connections at 6 months		-0.042**			-0.052**	
		(0.012)			(0.013)	
Unpaid work × personal connections		0.018			0.143*	
		(0.073)			(0.073)	
In STEM at 6 months			0.163**			0.514**
			(0.010)			(0.011)
Unpaid work × in STEM			-0.209			-0.235**
			(0.126)			(0.086)
Good Degree	0.206**	0.205**	0.193**	0.052**	0.051**	0.015
	(0.011)	(0.011)	(0.011)	(0.011)	(0.011)	(0.010)
Male	0.175**	0.176**	0.147**	0.137**	0.138**	0.058**
	(0.009)	(0.009)	(0.010)	(0.010)	(0.010)	(0.009)
High SES	0.081**	0.081**	0.081**	0.010	0.010	0.010
	(0.011)	(0.011)	(0.011)	(0.011)	(0.011)	(0.010)
Degree Class unclassified	-0.012	-0.011	-0.012	-0.018	-0.018	-0.021
	(0.033)	(0.033)	(0.032)	(0.031)	(0.031)	(0.027)
SES not classified	-0.017	-0.018	-0.019	0.029	0.028	0.020
	(0.015)	(0.015)	(0.015)	(0.015)	(0.015)	(0.014)
White	(Base)	(Base)	(Base)	(Base)	(Base)	(Base)
Black	0.045	0.042	0.047	-0.036	-0.039	-0.031
	(0.027)	(0.027)	(0.026)	(0.029)	(0.029)	(0.027)
Asian	0.086**	0.086**	0.086**	0.012	0.012	0.007
	(0.020)	(0.020)	(0.019)	(0.019)	(0.019)	(0.018)
Other	0.020	0.020	0.025	-0.038	-0.039	-0.021
	(0.021)	(0.021)	(0.021)	(0.021)	(0.021)	(0.018)

Continued on next page

Continued from previous page

	Outcome 3.5 years after graduation					
Dependent variables→	Real ln salary[1]			In STEM workforce		
	(1)	(2)	(3)	(4)	(5)	(6)
	Overall	By use of personal connect's	By initial STEM occupation	Overall	By use of personal connect's	By initial STEM occupation
2003 graduate	(base)	(base)	(base)	0.000	0.000	0.000
2005 graduate	0.083**	0.083**	0.071*	0.071*	0.072*	0.037
	(0.028)	(0.028)	(0.028)	(0.034)	(0.034)	(0.032)
2007 graduate	0.008	0.007	-0.016	0.089**	0.088**	0.011
	(0.029)	(0.029)	(0.029)	(0.034)	(0.034)	(0.031)
2009 graduate	-0.062*	-0.063*	-0.075**	-0.003	-0.004	-0.028
	(0.028)	(0.028)	(0.028)	(0.034)	(0.034)	(0.031)
N	10,170	10,170	10,170	8,300	8,300	8,300

Stars indicate: * p<0.05, ** p<0.01. Standard errors in parentheses. [1]Real ln salary regressions (models (1) – (3)) are Tobit regressions, values deflated to January 2013 pounds sterling, conditional on being in paid work. [2]Definitely STEM (models (4) - (6)) are OLS regressions, sample restricted to (i) those in paid or unpaid work 6 months after graduation and (ii) those in paid work with a salary or occupation coded from the survey 3.5 years after graduation.

Table C: Sample restricted to 'young' students (aged under 21 at university entry): Multivariate regression of whether six-month position was found through personal connections or was in STEM, on demographic and job characteristics.

Dependent variable: →	Six-month position found through personal connections			Six-month position was in a STEM field		
Sample→ Explanatory variables ↓	All workers	Unpaid workers only	Paid workers only	All workers	Unpaid workers only	Paid workers only
Unpaid Work	0.102**			-0.013*		
	(0.006)			(0.006)		
Female	-0.019**	-0.053**	-0.018**	-0.159**	-0.047**	-0.162**
	(0.002)	(0.013)	(0.002)	(0.002)	(0.012)	(0.002)
High SES	0.008**	0.009	0.008**	-0.002	0.011	-0.002
	(0.002)	(0.015)	(0.002)	(0.002)	(0.013)	(0.002)
Good Degree	-0.017**	0.017	-0.017**	0.090**	0.062**	0.091**
	(0.002)	(0.014)	(0.002)	(0.002)	(0.013)	(0.002)
SES not classified	-0.004	-0.021	-0.003	0.013**	0.007	0.013**
	(0.003)	(0.020)	(0.003)	(0.003)	(0.018)	(0.003)
Degree class not classified	-0.009	-0.030	-0.008	0.009	-0.066	0.010
	(0.007)	(0.064)	(0.007)	(0.007)	(0.058)	(0.007)
Ethnicity dummies	Yes	Yes	Yes	Yes	Yes	Yes
Cohort Dummies	Yes	Yes	Yes	Yes	Yes	Yes
N	173,260	4,870	168,390	172,995	4,820	168,170

* p < 0.05, ** p < 0.01. standard errors in parentheses. OLS regression: Dependent variable = 1 if found position taken six months after graduation using personal connections, 0 otherwise.

The results in this table are direct counterparts to the corresponding figures in Table 3 in the main body of the paper.

Table D: Restricted samples: No imputation for salaries, and young students only. Multivariate regression of labour market outcomes 3.5 years after graduation on demographic and job characteristics 6 months after graduation.

	Outcome 3.5 years after graduation					
	Excluded imputed salaries		Young students only			
Dependent variables→	Real ln salary[1]		Real ln salary[1]		In STEM workforce	
	(1) By use of personal connect's	(2) By initial STEM occupation	(3) By use of personal connect's	(4) By initial STEM occupation	(5) By use of personal connect's	(6) By initial STEM occupation
Unpaid work at 6 months	-0.227**	-0.182**	-0.238**	-0.180**	-0.101**	0.009
	(0.049)	(0.035)	(0.048)	(0.034)	(0.035)	(0.033)
Personal connections at 6 months	-0.034**		-0.047**		-0.048**	
	(0.012)		(0.013)		(0.013)	
Unpaid work × personal connections	0.028		0.062		0.158*	
	(0.078)		(0.075)		(0.075)	
In STEM at 6 months		0.149**		0.157**		0.509**
		(0.009)		(0.010)		(0.012)
Unpaid work × in STEM		-0.185		-0.207		-0.211*
		(0.137)		(0.144)		(0.095)
Good Degree	0.192**	0.181**	0.205**	0.194**	0.051**	0.015
	(0.011)	(0.011)	(0.012)	(0.011)	(0.011)	(0.010)
Male	0.157**	0.131**	0.171**	0.143**	0.136**	0.058**
	(0.009)	(0.010)	(0.010)	(0.010)	(0.011)	(0.010)
High SES	0.081**	0.080**	0.074**	0.074**	0.011	0.011
	(0.011)	(0.011)	(0.011)	(0.011)	(0.012)	(0.010)
Degree Class unclassified	-0.015	-0.016	-0.013	-0.012	-0.042	-0.034
	(0.032)	(0.032)	(0.037)	(0.036)	(0.034)	(0.031)
SES not classified	-0.010	-0.012	-0.030	-0.031	0.027	0.020
	(0.015)	(0.015)	(0.017)	(0.017)	(0.017)	(0.015)
Ethnicity dummies	Yes	Yes	Yes	Yes	Yes	Yes
Cohort dummies	Yes	Yes	Yes	Yes	Yes	Yes
N	9220	9220	9380	9380	7637	7637

Stars indicate: * p<0.05, ** p<0.01. Standard errors in parentheses. [1]Real ln salary regressions (models (1) – (3)) are Tobit regressions, values deflated to January 2013 pounds sterling, conditional on being in paid work. [2]Definitely STEM (models (4) - (6)) are OLS regressions, sample restricted to (i) those in paid

or unpaid work 6 months after graduation and (ii) those in paid work with a salary or occupation coded from the survey 3.5 years after graduation.

Columns 1 and 2, and 3 and 4, are counterparts to columns 2 and 3 in Table B.

Columns 5 and 6 are counterparts to columns 5 and 6 in Table B.

Table E: Characteristics of Science Graduates and Survey Respondents

	All science graduates	Science graduates with a first degree, responding to survey			
		All		Complete SES and Degree class information	
		6 month survey	3.5 year survey	6 month survey	3.5 year survey
	(1)	(2)	(3)	(4)	(5)
**Demographics:**					
High SES	27.8	33.3	36.2	55.3	56.9
Low SES	23.9	26.9	24.5	44.7	43.1
SES Not classified	18.8	16.5	14.8	x	x
SES Not collected	29.9	23.3	21.5	x	x
Male	53.2	51.6	48.8	50.4	47.6
Female	46.8	48.4	51.2	49.6	52.4
Good Degree	x	63.2	67.3	66.0	70.0
Lower Degree	x	34.3	30.3	32.0	28.0
Degree class unclass’	x	2.5	2.4	2.0	2.0
White British	80.9	83.2	79.4	86.1	84.5
Black Brit’ inc.mixed	3.1	2.6	3.7	2.1	2.3
Asian Brit’ inc.mixed	10.0	9.6	9.9	8.0	7.3
Other ethnicity	5.9	4.6	7.0	3.7	5.9
N	692,470	442,335	25,755	266,090	16,410
**Labour market outcomes 6 months after graduation:**					
Unpaid work	x	1.8	1.8	2.0	1.9
Paid work	x	63.7	60.8	63.5	60.0
Further study	x	19.9	23.4	20.1	24.5
Something else	x	14.5	14.0	13.9	13.6
N	692,470	442,335	25,755	266,090	16,410
Conditional on being in paid or unpaid work after 6 months, in known STEM/non-STEM field:					
Unpaid work	x	2.7	2.8	3.0	3.1
Paid work	x	97.3	97.2	97.0	96.9
Personal connections	x	19.0	19.4	19.2	19.5
STEM	x	25.1	29.7	23.7	28.7
Unpaid *and* Personal connections	x	0.8	0.8	0.9	0.9
Unpaid *and* STEM	x	0.6	0.7	0.6	0.8
Paid *and* Personal connections	x	18.2	18.6	18.4	18.7
Paid *and* STEM	x	24.5	29.0	23.1	28.2
N		275,020	16,110	162,560	10,150

Note: All 3.5 year survey respondents also participated in the 6 month survey. Degree class not included in the data extract for those not responding to the survey.

Table F: Labour market outcomes of science graduates 6 months after graduation

Sample → / Outcome ↓	All Science Graduates	High SES	Low SES	Women	Men	Good degree	Lower degree
	Percentage among:						
**Paid work**	63.72	62.069	65.252	64.162	63.306	61.641	67.284
Difference:		-3.183**		0.856**		-5.643**	
(Std. error)		(0.187)		(0.145)		(0.148)	
**Further Study**	19.92	22.201	18.598	21.038	18.874	23.692	13.453
Difference:		3.603**		2.164**		10.239**	
(Std. error)		(0.156)		(0.120)		(0.117)	
**Unpaid work**	1.84	2.109	1.941	2.193	1.502	1.931	1.672
Difference:		0.169**		0.691**		0.258**	
(Std. error)		(0.055)		(0.041)		(0.041)	
**Something Else**	14.52	13.62	14.209	12.608	16.319	12.736	17.59
Difference:		-0.589**		-3.711**		-4.854**	
(Std. error)		(0.135)		(0.105)		(0.113)	
N	442,335	147,065	119,029	213,900	228,430	279,395	162,935

Excludes those with unclassified SES or degree class. Stars indicate: * p<0.05, ** p<0.01
