# Supplementary material for: Unpaid work and access to science professions
Source: PLoS One. 2019 Jun 19;14(6):e0217032. doi: 10.1371/journal.pone.0217032 (PMC6583997; doi:10.1371/journal.pone.0217032)
Supplement: S1 Appendix — (PDF) [file pone.0217032.s007.pdf]

University of Essex

Research and Enterprise Office
T [redacted]
F
E
www.essex.ac.uk/reo

Colchester Campus
Wivenhoe Park
Colchester CO4 3SQ
United Kingdom
T [redacted]
F
www.essex.ac.uk

18 March 2015

[redacted]
Institute for Social and Economic Research
University of Essex

Dear [redacted]

Ethics Committee Decision

I am writing to advise you that your research proposal entitled '*Inequality in Higher Education Outcomes in the UK: subjective expectations, preferences, and access to information*' has been reviewed on behalf of the Social Science Faculty Ethics Sub-Committee and we are content to give a favourable ethical opinion of the research. I am pleased, therefore, to confirm that your application has been granted ethical approval by the University Ethics Committee.

Please do not hesitate to contact me if you require any further information.

Yours sincerely

[redacted]

Research Governance and Planning Manager

50% recycled material

1964
2014

The Queen's Anniversary Prizes
For Higher and Further Education
2009
