# Supplementary material for: Unpaid work and access to science professions
Source: PLoS One. 2019 Jun 19;14(6):e0217032. doi: 10.1371/journal.pone.0217032 (PMC6583997; doi:10.1371/journal.pone.0217032)
Supplement: S2 Appendix — (PDF) [file pone.0217032.s008.pdf]

HESA SERVICES LIMITED

95 Promenade
Cheltenham
GL50 1HZ

Tel: 01242 211133
Fax: 01242 211122
Web: www.hesa.ac.uk

Date: 28 February 2014
Ref: 34594_v4_AT

SCHEDULE TO THE AGREEMENT FOR THE SUPPLY OF DATA BETWEEN HESA SERVICES LIMITED AND THE CLIENT

IP4 (V 1.5) SCHEDULE OF SUPPLY

1.	Client:	The University of Essex
2.	Contact name:	[redacted]
3.	Client address:	Wivenhoe Park<br>Colchester<br>Essex<br>CO4 3SQ
4.	Client telephone:	[redacted]
5.	Client fax:	
6.	Billing address (if different from above):	N/A
7.	Delivery address (if different from above):	N/A
8.	Commencement Date:	1 May 2014
9.	Licence End Date:	31 December 2015
10.	Payment(s)(£) Excluding VAT:	[redacted]
11.	Payment(s) frequency:	
12.	Data:	All data to be supplied in tab de-limited text file format.

Item 1
All leavers 02/03 - 10/11 and DLHE longitudinal 2006/07 and 2008/09

Leavers population marker
Longitudinal leavers population marker (Where applicable)

Student fields
Unique identifier
Age *(17 and under, 18-20, 21-24, 25-29, 30-35, 36 and over, unknown)*
Gender
Ethnicity *(White / Black or Black British - Caribbean / Black or Black British - African / Other Black background / Asian or Asian British - Indian / Asian or Asian British - Pakistani / Asian or Asian British - Bangladeshi / Chinese / Other Asian background / Other, including mixed / Unknown)*
Nationality
Disability *(Disabled/No known disability)*
Level of study *(Postgraduate research/Postgraduate taught/First degree/Foundation degree/Other undergraduate)*
Low participation marker (Polar 3)
Domicile *(Sector Postcode)*
Institution *(HESA id and name)*
Location of Institution *(Sector postcode from 2005/06 only)*
Subject area - JACS 1.7 2002/03 to 2006/07
Subject area - JACS 2 2007/08 to 2010/11
Mode of study *(full-time/part-time)*
Highest qualification on entry *(XHQUAL01 for 2002/03 and 2009/10)*
Highest qualification on entry *(XQUALENT01 for 2010/11)*
Tariff score - *please note there are a number of comparability issues regarding this data between 2002/03 and 2010/11, these will be highlighted in the supporting documentation.*
First degree classification
Socio-economic Classification
Parental education (available from 07/08)
State school marker *(state school/private school/unknown N/A)*

DLHE fields
Method
April/January survey marker
Activity

Mode of further study
Employment circumstances (EMPCIR)
Duration
Standard Occupational Classification *(4 digit)*
Location of employment *(UK Sector postcode /Non-UK country)*
SIC (4 digit) - 2002/03 to 2006/07 (1992 version)
SIC (4 digit) - 2007/08 to 2010/11 (2007 version)
Employer size
Salary
How found job
Category of previous employment (PREVCAT 1-6)
Career codes (1-8, 02/03 – 06/07) (1-12, 07/08-11/12))
Importance to employer (EMPIMP)
Previously employed (PREVEMP)
Employed during course (EMPCRSE)
Qualification required for job (QUALREQ)
Teaching employment marker (TCHEMP)
Type of further study (NATSTUDY)
Type of qualification of further study (TYPEQUAL)

Item 1a:DHLE long fields (02/03 and 04/05)
Unique identifier
Main activity
Derived activity (pubcat)
Location of employment *(UK Sector postcode/ Non-UK country)*
Standard Occupational Classification (4 digit)
Standard Industrial Classification (4 digit)
Employer size
Employment status (Yes/No - derived PUBCAT)
Employment type (EMPTYPE)
Salary
Basis of employment (EMPSTATUS)
How did you find out about this job (02/03, FINDOUT 1-16, 04/05 Q14, 1-16)
Q12_1 Importance of qualification obtained (Factor1 02/03)
Q12_2 Importance of subject studied (Factor2 02/03)
Q12_3 Importance of degree classification (Factor3 02/03)
Q12_4 Importance of skills and competencies (Factor4 02/03)
Q12_5 Importance of additional qualifications (Factor5 02/03)
Q12_6 Importance of work experience (Factor6 02/03)

Q3: Date started job
Q4: Employment type
Q7: Country of employment
Number of jobs
Have you ever been unemployed
Number of periods of unemployment
Have you obtained any other qualifications
What was the highest level of qualification you obtained (QUALS_HLEV)
Likelihood of doing a different subject? (DIFSUB)
Likelihood of studying at a different institution? (DIFINST)
Likelihood of working towards a different type of qualification (DIFQUAL)
Career satisfaction (SATIS)
Weighted FPE
Unweighted FPE

Item 1b: DLHE long fields (06/07 and 08/09)
Unique identifier
Main activity on snapshot date (ACT_MAIN)
Derived activity (pubcat)
Location of employment *(UK Sector postcode/ Geographical Region)*
Standard Occupational Classification (4 digit)
Standard Industrial Classification (4 digit)
Employer size
Employment type (HOW_EMP)
Derived salary verified (WRK_SAL_VRFD)
Basis of employment (WRK_BASIS)
How did you first find out about this job? (HOW_FND_JOB)
Employment start date
Q3: Working in more than one job
Q4: How many jobs
Q7: Employer name
Q12: Basis of employment
Q16_1 Importance of subject studied (IMPO_SUB)
Q16_2 Importance of qual obtained (IMPO-JOB)
Q16_3 Importance of qual grade (IMPO_GRDE)
Q16_4 Importance of skills (IMPO_SKLS)
Q17_1 Importance of additional quals
Q17_2 Importance of...Relevant work experience
Q25: Qualification aim
Q31: Have you had other jobs since graduating

Q32: How many other jobs
Q33: Have you ever been unemployed
Q34: Number of periods of unemployment
Q35: Total months unemployed
Q36: Have you obtained any other qualifications since 06/07
Q38 What was the highest level of qualification you obtained (QUALS_HLEV)
Q41_1 Likelihood of doing a different subject? (LK_DIF_SUB)
Q41_2 Likelihood of studying at a different institution? (LK_DIF_INST)
Q41_3 Likelihood of working towards a different type of qualification (LK_DIF_QUAL)
Q42 Career satisfaction (CAREER_SAT)
Q43 Good value for money (QUALS_EXP)
Weighted FPE
Unweighted FPE

Item 2
All leavers from UK HE institutions 2011/12

Student fields
Age *(17 and under, 18-20, 21-24, 25-29, 30-35, 36 and over, unknown)*
Gender
Ethnicity *(White / Black or Black British - Caribbean / Black or Black British - African / Other Black background / Asian or Asian British - Indian / Asian or Asian British - Pakistani / Asian or Asian British - Bangladeshi / Chinese / Other Asian background / Other, including mixed / Unknown*)
Nationality
Disability *(Disabled/No known disability)*
Level of study *(Postgraduate research/Postgraduate taught/First degree/Foundation degree/Other undergraduate)*
Low participation neighbourhood (POLAR3)
Domicile *(Sector Postcode)*
Institution *(HESA id and name)*
Location of Institution *(Sector postcode)*
Subject area
Mode of study *(full-time/part-time)*
Highest qualification on entry (QUALENT2)
Highest qualification on entry *(QUALENT3)*
Tariff
First degree classification

Socio-economic Classification
Parental education
State school marker *(state school/private school/unknown N/A)*

DLHE fields
Status of data collection
April/January survey marker
Activity (1-8)
Mode of further study
Most important activity (MIMPACT)
Employment activity (XACTIV02)
Reasons for taking the job (JOBRSNALL1-9)
Main reason for taking the job (JOBRSNMAIN)
Employment basis (EMPBASIS)
How found job (JOBFOUND)
Importance to employer (EMPIMP)
Number of jobs JOBSNO,
Part-time hours per week (PTHOURS)
Place of employment (EMPLACE)
Postdoctoral research contract (POSTDOC)
Previously employed (PREVEMP)
Category of previous employment (PREVWORK1-9)
Qualification required for job (QUALREQ)
Salary
Standard Occupational Classification (SOCDLHE2010,)
Total estimated earnings (ESTERN)
Location of employment (EMPPCODE)
Unpaid work (EMPUNPAID)
Type of qualification (TYPEQUAL)
Employed as teacher (EMPLDTEACH)
Employer size
SIC (4 digit)
HE experience for business (Q31)
HE experience for study (Q30)
HE experience for work (Q29)

Item 3: First Destinations datasets 1994/95 to 2001/02:

Student fields:
Age *(17 and under, 18-20, 21-24, 25-29, 30-35, 36 and over, unknown)*
Gender
Ethnicity 2001/02 *(White / Black or Black British - Caribbean*

/ Black or Black British - African / Other Black background / Asian or Asian British - Indian / Asian or Asian British - Pakistani / Asian or Asian British - Bangladeshi / Chinese / Other Asian background / Other, including mixed / Unknown)
Ethnicity 1994/95 to 2000/01 (*White, Black Caribbean, Black African, Black other, Indian, Pakistani, Chinese, Asian other, Other, unknown*)
Nationality (*where supplied, field not compulsory in early years*)
Disability *(Disabled/No known disability)*
Level of study *(Postgraduate research/Postgraduate taught/First degree/Foundation degree/Other undergraduate)*
Domicile *(Sector Postcode)*
Institution *(HESA id and name)*
Location of Institution *(Sector postcode)*
Subject area - HESACODE
Mode of study *(full-time/part-time)*
Highest qualification on entry (grouped)
Degree classification

First destination fields:
Main activity 1 and 2
Duration of employment
Employment category
Location of employment (full)
Mode of employment
Not available for employment
Professional subject of training
SIC
SOC
Employer sponsorship
Nature of study

13. Expected timescale for delivery (from receipt of this signed Schedule): 30-35 Working days

14. Permitted Purposes: Data may only be used for academic research projects: [redacted] 'Inequalities in Higher Education outcomes' [redacted]

All analysis of DLHE Longitudinal data must be calculated using the weighted FPE, although data suppressions must be applied to the unweighted FPE. See standard rounding methodology for further details.

DLHE Longitudinal data must not be published at institution level.

Any data published within the reports must be rounded to the nearest 5 according to the standard rounding methodology as outlined in special condition 1.

15. Special conditions (if any):

1. Any reproduction or publishing of Data by the Client, subject to the above Permitted Purposes, must adhere to the HESA Services Standard Rounding Methodology – see Terms and Conditions Clause 7.3.
2. All copies of Data must be destroyed by the Licence End Date shown above – see Clause 6.6.
3. Ethnicity data is provided for the purposes of promotion and maintenance of Equal Opportunities.
4. Ethnicity and disability data may not be cross-tabulated together in any analyses/extracts passed to a third party.
5. Access to the raw data must be restricted to [redacted] Angus Holford, [redacted]. Appropriate technical means must be established to prevent unauthorised access to the raw data.
6. The data, or extracts thereof, may only be reproduced in a research paper(s) or research report(s). Special permission is required before any HESA data is reproduced on an Internet Web-site.
7. No data may be passed to any third party without the written permission of HESA Services Ltd.
8. The data may not be used for institutional planning or promotion purposes.
9. The data may not be used for teaching purposes.
10. The data may not be used to generate commercial income in any form.
11. The data must not be used to identify individuals. No data may be reproduced in any form that would allow a third party to identify or derive information

HESA Services Ltd is registered in England at the above address. Registered No. 3109219. The company is a wholly owned subsidiary of the Higher Education Statistics Agency Ltd (Registered in England No. 2766993. Registered Charity No. 1039709). Certified to ISO 9001 and ISO 27001.

about individuals. If there is any doubt on this issue HESA Services Ltd. must be consulted.

12. Data must not be passed to independent or commercial consultants.
13. The final paper(s) or report(s) must acknowledge HESA or HESA Services Ltd., as the source of data, using a form of words specified by HESA Services Ltd.
14. A copy of the final paper or report must be sent to HESA Services Ltd., as soon as is reasonably practicable.

16. Publication caveats/data attribution:	Source:<br>HESA Destination of Leavers survey 2002/3 -2011/12<br>HESA Destination of Leavers Longitudinal survey 2006/07 and 2008/09<br>HESA First Destinations record 1994/95 to 2001/02<br>Copyright Higher Education Statistics Agency Limited 2013<br>HESA cannot accept responsibility for any inferences or conclusions derived from the data by third parties.

This document is the Schedule to the Agreement for the Supply of Data, a copy of which has been supplied to the Client (the "Agreement"). In signing this Schedule, the parties are agreeing to the terms and conditions set out in the Agreement, including this Schedule.

Signed by :
for the Client ....

Name: ....

Date: ....

Signed by :
for HESA Services Limited ....

Name: ....

Date: ....
